# Cytomorphological patterns of breast lesions among women with palpable breast lumps attending select teaching and referral hospitals in Kenya: a descriptive cross-sectional study

**DOI:** 10.11604/pamj.2023.44.171.37755

**Published:** 2023-04-13

**Authors:** Josephine Nyabeta Rioki, Lucy Muchiri, Marshal Mweu, Elijah Songok, Emily Rogena

**Affiliations:** 1 Department of Human Pathology, Faculty of Health Sciences, University of Nairobi, Nairobi, Kenya,; 2 Department of Public and Global Health, Faculty of Health Sciences, University of Nairobi, Nairobi, Kenya,; 3 Kenya Medical Research Institute (KEMRI), Nairobi, Kenya,; 4 Department of Pathology, Jomo Kenyatta University of Agriculture and Technology, Kiambu, Kenya

**Keywords:** Breast lesions, fine needle aspiration cytology, women, International Academy of Cytology, palpable breast lumps

## Abstract

**Introduction:**

breast lumps account for a greater number of lesions in women attending surgical clinics in the developing world. Breast cancer which mostly presents as a breast lump is the leading cancer in Kenya, with an incidence of 12.5%. The study aims to describe the patterns of breast lesions in women presenting with palpable breast lumps in two major referral hospitals in Kenya.

**Methods:**

seven hundred and sixty-eight study participants with palpable lumps underwent fine needle aspiration cytology (FNAC). Sociodemographic data were captured using structured questionnaires. The FNAC materials were evaluated using the International Academy of Cytology Yokohama System (IACYS) and the lesions were classified into five-tier categories. Frequencies and percentages were used to summarize qualitative variables.

**Results:**

of 768 smears, 84.8% (n=651) were adequate for evaluation while 15.2% (n=117) were inadequate. Neoplastic lesions comprised 84.5% (n=550) and non-neoplastic 15.5% (n=101). Benign lesions accounted for 83.6% of the lesions followed by breast carcinoma (10.4%). Ductal carcinoma comprised 98.5% of cancerous lesions. The age group most affected with ductal carcinoma and suspicious lesions was 20-34 years (37.3% and 55.6% respectively). Fibroadenoma formed the bulk of the benign lesions identified (44.1%). Suspicious of malignancy was 4.1% (n=27). The age group with the most lesions (47.5%) was 20-34 years.

**Conclusion:**

a wide spectrum of breast lesions was established. Such include inflammatory, atypical, benign, suspicious of malignancy, and malignant lesions. Fibroadenoma was a common lesion diagnosed. The age group most affected by malignant lesions was 16-49 years, necessitating enhanced screening of women with breast lumps in our setups.

## Introduction

Breast cancer which mostly presents as a lump on the breast is leading in Kenya, with an incidence of 12.5% and a prediction of an increase to 35% in 2025 [1]. Breast lumps encompass both benign and malignant lesions. The lesions usually present as palpable lumps [2]. Breast lumps account for a greater number of lesions in women presenting to surgical clinics in the developing world [3]. The different categories of lesions of the breast as per the International Academy Of Cytology (IAC) [4] classification include C1-C5 (insufficient, benign, atypical, suspicious of malignancy, and malignant lesions) [5,6]. The triple test approach (physical examination, radiology, and cytopathology) is used in the evaluation of breast lesions. Fine needle aspiration cytology (FNAC), one of the “triple test” approaches for evaluating breast lumps distinguish neoplastic and non-neoplastic lesions of the breasts [7,8]. This technique is rapid, reliable, highly acceptable by patients, cost-effective, and has low complication rates [9,10]. Globally, FNAC has been used to guide the diagnosis and follow-up of patients on treatment for breast cancer [11,12]. It can be of use in advanced carcinoma or in patients not willing to undergo surgery [13]. Generally, this may form the basis of management in such cases.

In Kenya, FNAC has been the preferred procedure as far back as the late nineties, especially at the Kenyatta National Hospital (KNH). However, in the most recent times, due to changes in management guidelines for breast cancer, there has been a shift to the use of core biopsies, even though, FNAC is still practiced in many health facilities in Kenya, especially in remote areas. With the emerging trend of breast cancer and limited resources, patients with breast lumps are prone to develop a lot of anxiety. The waiting time for tissue diagnosis is long, and the cost implication further aggravates the situation. This, therefore, calls for screening of such cases using FNAC to establish whether the lesions are benign or malignant prior to definite treatment. Furthermore, its utilization will alleviate anxiety in patients, reduce the burden of testing, and as well enhance the rapid management of patients.

Due to human (pathologists) resource scarcity in Kenya, task sharing and shifting training was carried out in 2016 [14] to ensure diagnostic pathology services were offered to all Kenyans at affordable rates and timely diagnosis ensued. This coincidentally served as sensitization to the utilization of FNAC in the screening and diagnosis of palpable lumps. This is evidenced by the increase in the number of patients who were screened soon after the training. An increase in fine needle aspiration biopsy by 41% [14] was noted after the training. Since there is high demand for diagnostic services and vast experience gained by physicians in the country, there is a likelihood of detecting emerging patterns of breast lesions in Kenya. Despite this, little is known about the overall disease distribution patterns. Considering this, a study was conducted in two major referral hospitals to describe the pattern of the lesions of the breast among women with palpable lumps of the breast. This serves to inform the clinicians and other stakeholders on the spectrum of breast lesions for effective screening and diagnostic plans in our setups.

## Methods

**Study design and setting:** this was a cross-sectional descriptive study, involving female patients who presented to FNA/surgical clinics in Nakuru Provincial General Hospital (NKPGH) and Kenyatta National Hospital (KNH), Kenya between December 2016 to December 2018. This work is part of a bigger study, reference number P334/04/2016 whose main objective was to characterize breast cancer phenotypes and genotypes among women diagnosed with breast cancer at the selected teaching and referral health facilities.

**Study participants:** women with palpable breast lumps with no prior diagnosis of breast cancer, aged 16 to 97 years, and with written consent were considered eligible to participate in the study.

**Sample size estimation:** the sample size was calculated using Fisher´s formula [15]. A confidence interval of 95% and 5% degree of precision were applied. Since the prevalence of breast lesions on cytology in our setups is not known, a proportion of 50% was used. A minimum sample size of 384 was deduced and applied to each study site (KNH and NKPGH). Overall, 768 women were enrolled in the study.

**Recruitment of study participants:** participants were recruited from outpatient fine needle aspirate/surgical clinics. The study was introduced to all female patients as they walked into the clinics by a researcher/research assistant. The benefits and risks of the study were explained. Those who accepted to participate in the study were given consent forms to sign. Those who were not able to write were assisted by their guardians. After consenting, the researcher/research assistant administered the questionnaires and at this time, the patients´ history to ascertain if they had a prior diagnosis of breast cancer or trauma was also taken. All patients who consented were involved in the study on a first come first basis till the allotted sample size was achieved.

### Specimen collection, processing, and reporting

**Fine needle aspiration procedure, smear preparation, and staining:** a pathology registrar/research assistant were involved in collecting the fine needle aspirate material. Briefly, palpation of the breast lump was made, and after estimating the size, the skin was sterilized using surgical spirit swabs. The lump was then localized and fixed firmly between the first digit of the hand and the index finger of the non-aspirating hand. Using a 21-gauge needle and five or ten milliliters disposable syringe from Becton and Dickinson (BD) attached, the plunger of the syringe pistol was drawn to create negative pressure [16,17]. Steadily, the needle was inserted through the skin, directly to the lump. Three to four passes were made through chisel-like movements before the needle was withdrawn. The FNA material was put onto the glass slides by pressing the material from the needle hub. Another slide used as a spreader was placed on top of the material and the two were pulled apart making a uniform smear on the prelabeled slide. For each patient, 3 smears were prepared. One of the smears prepared was air dried and stained with the May Grunwald Giemsa (MGG) staining technique while the other two were fixed in 95% ethyl alcohol for 15 minutes and stained using Hematoxylin and Eosin and Papanicolaou stains.

**Reporting of the smears:** the stained slides/smears were reported within the Department of Human Pathology, University of Nairobi. The slides were scanned using low power at X4 to evaluate specimen adequacy and X10 for screening the smears using an Olympus microscope. The identified lesions were categorized into a five-tier using a reporting format for breast lesions described/recommended by The International Academy of Cytology (IAC) Yokohama in 2016, as, inadequate/insufficient material (C1), benign (C2), atypical (C3), suspicious of malignancy (4), and malignant (C5) [18].

**Data collection and analysis:** we used structured questionnaires to collect sociodemographic information such as the age and location of the participants among others. For laboratory analysis, we made observations on stained smears to differentiate the breast lesions, whose frequencies were calculated. Analysis of data was done using Statistical Package for the Social Sciences (SPSS) Version 19. Categorical data (age groups and diagnostic categories) were analysed as counts and percentages using data tables and the results were presented using two-way tables.

**Ethical approval:** this study was approved by the Kenyatta National Hospital-University of Nairobi Ethical Review Committee (KNH-UoN ERC); study number P 334/16 and institutional review boards of the select facilities.

## Results

**Study participant characteristics:** a total of seven hundred and sixty-eight (768) women with breast lumps presenting to FNA clinics were sampled consecutively and FNA materials were collected. The study participants' age ranged from 16-97 years. Most of the study participants (47%; n=307) were between the age bracket of 20-34 years. Those in the age range of 35-49 years were 26% (n=171), followed by 81 in the ≤ 20 years category (12%), 11% (n=71) in the 50-64 years category, 3% (n=16) in 65-79 years category and 1% (n=4) above 80 years. Of the 768 FNA material aspirated, 117 (15.2%) were inadequate (insufficient) for reporting while 651 (84.8%) were adequate for evaluation. Inadequate samples were not included in the study.

**Classification of breast lesions:** using a five-tier International Academy of Cytology Yokohama System (IACYS), the lesions were reported and categorized as, insufficient/inadequate (C1) 15.2% (n=117), benign (C2) 83.6% (n=544), atypical (C3) 1.8% (n=12), suspicious of malignancy (C4) 4.1% (n=27) and malignant (C5) 10.4% (n=68) (Table 1).

**Table 1:** classification of the cytological diagnosis of breast lesions using IACYS (n=768)

Diagnostic categories		Diagnosis	Number of cases	Percentage (%)	Overall percentage (%)
C1	Insufficient	-	117	15.2	15.2
C2	Benign	Abscess	39	6	83.6
		Benign cyst	33	5.1	
		Benign proliferative lesions	48	7.4	
		Chronic granulomatous mastitis	3	0.5	
		Duct ectasia	24	3.7	
		Galactocele	32	4.9	
		Fat necrosis	3	0.5	
		Fibroadenoma	287	44.1	
		Fibrocystic change	29	4.5	
		Inflammatory lesions	24	3.7	
		Inflammatory lymph node	8	1.2	
		Lipoma	4	0.6	
		Normal breast tissue	10	1.5	
C3	Atypical	-	12	1.8	1.8
C4	Suspicious of malignancy	-	27	4.1	4.1
C5	Malignant	Breast carcinoma	68	10.4	10.4

IACYS: International Academy of Cytology Yokohama System; C: category

The breast lesions detected were classified as neoplastic lesions comprising 84.5% (n=550) and non-neoplastic lesions comprising 15.5% (n=101) of all diagnosed breast lesions. Overall, the benign lesions from the study were 544 translating to 83.6%. The neoplastic lesions comprised of benign, atypical, suspicious of malignancy, and malignant lesions.

**Distribution of IACYS diagnostic categories in relation to age groups:** the age group with the most malignant lesions was 20-34 years (36.8%), followed by 35-49 years (30.95%) and 50-64 years (19.1%), while the benign lesions were common in the group between 20-34 years (47.8%) followed by 25.6% in the 35-49 age group. In the 20-34 age bracket, the suspicious cases were 55.6% and 29.6% were in the 35-49 age bracket. The same case applies to the atypical category, 58.3% and 25% respectively (Table 2).

**Table 2:** frequencies of diagnostic categories among age groups (n=651)

Age group	Malignant	Suspicious	Atypical	Benign	Total
≤ 20	2(2.9%)	0(0%)	0(0%)	79(14.5)	81
20 - 34	25(36.8%)	15(55.6%)	7(58.3%)	260(47.8%)	307
35 - 49	21(30.9%)	8(29.6%)	3(25%)	139(25.6%)	171
50 - 64	13(19.1%))	2(7.4%)	2(16.7%)	55(10.1%)	72
65 - 79	5(7.4%)	2(7.4%)	0(0%)	9(1.7%)	16
≤ 80	2(2.9%)	0(0%)	0(0%)	2(0.4%)	4
Total	68(100%)	27(100%)	12(100%)	544(100%)	651

**Overall distribution of specific breast lesions according to specific diagnosis and age groups:** overall, the most common (44.1%) benign lesion identified in this study was fibroadenoma. Among the participants with fibroadenoma, the age group with these lesions was 20-34 years (56.8%), followed by 35-49 years (24.4%) and <20 years (18.8%). In those with ductal carcinoma, the age bracket with most lesions was 20-34 years (37.3%) followed by 35-49 years (31.3%). Malignant phyllodes was seen in only one case in the ≤ 20 years category. Breast lesions were less frequent above 65 years of age (Table 3).

**Table 3:** overall distribution of specific breast lesions in different age groups (n=651)

Diagnosis on cytology	Age group					
	Less than 20 years	20 - 34 years	35 - 49 years	50 - 64 years	65 - 79 years	80+ years
Atypia	0	7(58.3%)	3(25%)	2(16.7%)	0	0
Abscess	5(12.8%)	16(41%)	12(30.8%)	6(15.4%)	0	0
Benign cyst	0	12(36.4%)	11 (33.3%)	7(21.2%)	3(9.1%)	0
Benign proliferative lesions	7(14.6%)	26(54.2%)	11(23%)	2(4.2%)	1(2.1%)	1(2.1%)
Chronic granulomatous mastitis	1(33.3%)	0	2(66.7%)	0	0	0
Duct ectasia	0	9(37.5%)	6(25%)	7(29.1%)	2(8.3%)	0
Ductal carcinoma	1(1.5%)	25(37.3%)	21(31.3%)	13(19.4%)	5(7.5%)	2(3%)
Fat necrosis	0	0	0	1(33.3%)	1(33.3%)	1(33.3%)
Fibroadenoma	54(18.8%)	163(56.8%)	70(24.4%)	0	0	0
Fibrocystic change	0	0	5(17.2%)	24(82.8%)	0	0
Galactocele	1(3.1%)	14(43.8%)	12(37.5%)	5(15.6%)	0	0
Inflammatory lesions	3(12.5%)	12(50%)	6(25%)	1(4.2%)	2(8.3%)	0
Inflammatory lymph node	1(12.5%)	5(62.5%)	2(25%)	0	0	0
Lipoma	0	1(25%)	1(25%)	2(50%)	0	0
Normal breast tissue	7(70%)	2(20%)	1(10%)	0	0	0
Suspicious of malignancy	0	15(55.6%)	8(29.6%)	2(7.4%)	2(7.4%)	0

## Discussion

In this study, the establishment of the spectrum of breast lesions on FNAC among symptomatic patients presenting to FNA clinics in two major referral hospitals in Kenya was sought. Neoplastic lesions were 84.5% and 15.5% were non-neoplastic. Though neoplastic findings were comparable to those of Chaudhary *et al*. (81.9% and 79.5%), the number of non-neoplastic findings was inconsistent (3.6% and 24.1%) [3,19]. Fibroadenoma was the most commonly diagnosed neoplastic lesion followed by breast carcinoma. Similar findings were reported elsewhere [16]. Findings from this study indicate the occurrence of malignant breast lesions in women between 16-49 years, a much younger age group compared to the rest of the world.

**Malignant lesions:** from this study, 10.4% of the lesions were malignant. This is consistent with that of Gupta *et al*. (11%) [20] and Pangotra *et al*. (10.06%) [21]. However, these findings contradict those found by Nkonge *et al*. (27.7%) [19]. It has been shown in this study that ductal carcinoma was the most common (98.5%) breast cancer identified. These findings are consistent with those of other researchers in the literature [9,12,20,22,23]. One case of malignant phyllodes in the <20 years of age category was detected, a similar finding was obtained from another study [24].

In the present study, malignant lesions were common in the age group 20-34 years, followed by 35-49 years, findings that differ from other studies [9,13,25]. According to Bukhari, women in their 4^th^, 5^th^, and 6^th^ decades of life were the most affected with ductal carcinoma [12] while Bhatnagar *et al*. indicated that most malignant lesions occurred between 31 to 40 years, and 41 to 50 years [26]. Our findings show overall that women of the younger age group are the ones at risk of developing breast cancer. However, the risk of malignancy may have to be calculated to verify this fact.

**Benign lesions:** a predominance (83.6%) of benign lesions in the two health facilities was observed. These findings were comparable to those of Gupta *et al*. (89%) and Qadir *et al*. (88%) [20,27] but higher than those of Elmadhoun *et al*. (72%) [28]. Similar findings from other studies have been documented. The benign-to-malignant ratio in this study was about three times higher (8: 1) compared to a previous study (2.6: 1) by Nkonge *et al*. [19,28]. This could be due to the larger sample size (n=768) used in this study compared to the previous study (n=390). Of the benign lesions in our study, fibroadenoma was the most common (41%). This is consistent with the findings of Embaye *et al*. (40%) [8] but relatively lower (53.61%, and 56.2%) than that observed in other studies [3,9,23]. On the contrary, fibrocystic disease was the most common detected by Kumar [29]. In the present study, the inflammatory lesions were 15.6%, a comparable finding to that of Pangotra *et al*. [21] despite a small sample size compared to our study.

**Lesions suspicious of malignancy and atypical lesions:** in the present study, 4.1% of suspicious lesions of malignancy were diagnosed. These findings are comparable to those obtained by Priyanka *et al*. [23, 27]. However, lower (0.3%) and higher numbers (12%) have been described by Pangotra *et al*. respectively [21,30]. Atypical lesions from our study were 1.8%. These findings are slightly higher than those found by Pangotra *et al*. and Priyanka *et al*. [21,23].

**Inadequate material:** in this study, we observed a higher rate (15.2%) of inadequate smears. This rate compares with the baseline rate (18%) established by task sharing and shifting training in Kenya. The higher rates were also reported by the end of the task-sharing project in Nyeri Provincial General Hospital (NPGH) (16%) and Kisii Teaching and Referral Hospital (KTRH) (19%) [14]. Such findings could be due to a lack of supervision or inadequate training. Adequate training and supervision of the medical doctors, pathologists, and clinical officers yielded decreased inadequate rates to comparable rates (4%) across the literature. Comparable results (13.6% and 14.5%) were also established by Chaudhary and Bajwa *et al*., respectively [3,31]. This difference could be due to the sample size difference or that the specimens were collected by experienced physicians/pathologists.

**Diagnosed lesions with age-specific distribution:** with respect to age, it was established that women in the age range 20-34 years had more diagnosed breast lesions (47.2%), encompassing both benign and malignant lesions. Similar findings have been documented in India [4]. Findings from other studies have also revealed consistent findings of maximum cases occurring in the age range of 21-30 years [9,15,20,23]. However, contrary findings have been documented in Bangladesh where the age group with the most lesions were those between 12-20 years [5] and in Kenya, 20-24 years by Nkonge *et al*. [19]. The age group with the least lesions was those over 65 years. These findings are consistent with those reported by Manasa *et al*. [16].

Though evaluation of breast lumps is done by clinical examination, radiology, and FNAC/core biopsy, in this study, mammography/ultrasound reports were not available for most patients. However, the few cases with ultrasound reports available correlated well with FNAC.

**Unanswered questions and future research:** a study to correlate radiology findings with FNAC as well as the inclusion of rapid onsite evaluation (ROSE) in the evaluation of breast lumps, should be considered to limit the number of inadequate rates.

## Conclusion

A wide spectrum of breast lesions was established. Such include inflammatory, atypical, benign, suspicious of malignancy, and malignant lesions. Fibroadenomas were common benign neoplastic lesions diagnosed followed by breast carcinoma. The age group most affected by malignant lesions was 16-49 years, a much younger age group compared to the rest of the world. This calls for enhanced screening of young women for breast cancer in our setups. Since most lumps of the breasts are benign, FNAC can be used for their evaluation, especially in the most remote settings.

### What is known about this topic

- *Breast lesions encompass both benign and malignant tumors;*
- *Fine needle aspiration cytology is a rapid, cost-effective, and widely accepted method of evaluating breast lumps;*
- *In experienced hands, FNAC yields reliable results*.

### What this study adds

- *Breast lumps are common in women presenting to surgical clinics in Kenya; most of the lumps are benign and fibroadenoma is the most diagnosed lesion;*
- *The age group with the most lumps was 20-34 years;*
- *Young women in our setup are affected with malignant lesions; the age group most affected with malignant lesions is 20-34 years followed by 35-49 years*.
